# Integrating random walk and binary regression to identify novel miRNA-disease association

**DOI:** 10.1186/s12859-019-2640-9

**Published:** 2019-01-28

**Authors:** Ya-Wei Niu, Guang-Hui Wang, Gui-Ying Yan, Xing Chen

**Affiliations:** 1 School of Mathematics, Shandong University, Jinan, 250100 China; 2 Academy of Mathematics and Systems Science, Chinese Academy of Sciences, Beijing, 100190 China; 3 School of Information and Control Engineering, China University of Mining and Technology, No.1, Daxue Road, Xuzhou, 221116 Jiangsu China

**Keywords:** microRNA, Disease, miRNA-disease association, Random walk, Binary regression

## Abstract

**Background:**

In the last few decades, cumulative experimental researches have witnessed and verified the important roles of microRNAs (miRNAs) in the development of human complex diseases. Benefitting from the rapid growth both in the availability of miRNA-related data and the development of various analysis methodologies, up until recently, some computational models have been developed to predict human disease related miRNAs, efficiently and quickly.

**Results:**

In this work, we proposed a computational model of Random Walk and Binary Regression-based MiRNA-Disease Association prediction (RWBRMDA). RWBRMDA extracted features for each miRNA from random walk with restart on the integrated miRNA similarity network for binary logistic regression to predict potential miRNA-disease associations. RWBRMDA obtained AUC of 0.8076 in the leave-one-out cross validation. Additionally, we carried out three different patterns of case studies on four human complex diseases. Specifically, Esophageal cancer and Prostate cancer were conducted as one kind of case study based on known miRNA-disease associations in HMDD v2.0 database. Out of the top 50 predicted miRNAs, 94 and 90% were respectively confirmed by recent experimental reports. To simulate new disease without known related miRNAs, the information of known Breast cancer related miRNAs was removed. As a result, 98% of the top 50 predicted miRNAs for Breast cancer were confirmed. Lymphoma, the verified ratio of which was 88%, was used to assess the prediction robustness of RWBRMDA based on the association records in HMDD v1.0 database.

**Conclusions:**

We anticipated that RWBRMDA could benefit the future experimental investigations about the relation between human disease and miRNAs by generating promising and testable top-ranked miRNAs, and significantly reducing the effort and cost of identification works.

**Electronic supplementary material:**

The online version of this article (10.1186/s12859-019-2640-9) contains supplementary material, which is available to authorized users.

## Background

MicroRNAs (miRNAs) are one category of small single-stranded non-coding RNA molecule (containing 20~25 nucleotides), which function in regulation of gene expression at the posttranscriptional level [1, 2]. Generally, miRNAs could cause mRNAs degradation by binding to the 3′ untranslated regions (UTRs) of their target mRNAs [1–5]. Since the first discovery of miRNA about 20 years ago, a plenty variety of miRNAs have been discovered so far, ranging from nematodes to humans [6–10]. With the in-depth biology research about miRNAs, a vast amount of studies have explicitly shown that miRNAs played important roles in many fundamental biological processes, such as cell growth, proliferation, metabolism, differentiation, apoptosis, signal transduction and so forth [11–15]. In last decades, it was found that the dysregulation of miRNAs could lead to many maladjusted cell behaviors [16], which made miRNAs increasingly be recognized as key regulatory players in gene expression process. Therefore, it’s interpretable that many miRNAs have been reported to be related with the development of enormous complex human diseases, including cancers, neurological disorders and so on [17–19]. For example, Kliese et al. found that miRNA-145 was downregulated in atypical meningiomas and negatively functioned by regulating the proliferation and motility of meningioma cells [20]. Besides, it was found that in the breast cancer patient, the expression level of miRNA-141 was significantly higher than normal group [21]. What’ s more, Zhao et al. discovered that miRNA-106a could be seen as an independent biomarker in glioblastoma patients [22]. In addition, compared with normal lymph cells, the expression level of miRNA-19a in canine lymphoid cell lines was obviously increased [23]. Therefore, it’s meaningful and uncontroversial to regard disease-related miRNAs as potential biomarkers, which could not only significantly contribute to comprehending the diseases mechanisms, but also benefit the detection, prognosis, diagnosis, treatment and prevention of human complex diseases [24–27]. Nevertheless, the intrinsic disadvantage of traditional experimental method made the identification process of disease-miRNA associations costly and time consuming. Considering the massive increases in the reliability and volume of miRNA-related data based on the accumulated researches about miRNAs, it became necessary and doable to develop effective computational models for predicting potential miRNA-disease associations, which could further enhance the understanding of disease development in miRNA level. More importantly, the promising prediction results of computational approaches could also offer convenience for the follow-up validation experiment by biologic or biomedical researchers [28, 29].

Indeed, having the verified miRNA related data in one hand and the assumption that functionally similar miRNAs are more likely to be associated with phenotypically similar diseases and vice versa in the other, many computational methods have been proposed to predict the underlying miRNA-disease associations in aspect of network science, combinatorial optimization, machine learning, system biology and so on [9, 30–38]. For example, Jiang et al. [24] proposed a computational model based on hypergeometric distribution to predict novel miRNA-disease associations. They firstly constructed some classic network models, such as disease phenotypical similarity network, miRNA functional similarity network and known phenome-miRNAome network according to multi-source biological data. Then they integrated all the networks to finally prioritize the human miRNAs for diseases of interest. However, the strong dependence on the miRNA-target interactions resulted in a high rate of false positive result of the method. Xu et al. [39] investigated the expression profiles of miRNAs and proposed a computational model, in which they constructed miRNA target-dysregulated network to extract pivotal feature vectors for miRNAs. Support vector machine (SVM) was then conducted in their model to distinguish positive disease-related miRNAs from negative ones. However, the difficulty of obtaining negative disease-related miRNAs made the model have very narrow applications. Differing from traditional local network similarity measures, Chen et al. [40] utilized the global network similarity measures and proposed the Random Walk with Restart for MiRNA–Disease Association (RWRMDA) model. In this model, they constructed the global miRNA functional similarity network, on which they further implemented random walk with restart. Based on the stationary state of the random walk dynamic process, namely the association probability of each disease-miRNA pair, authors finally prioritized candidate miRNAs for diseases investigated. Likewise, focusing on the functional connections between miRNA targets and disease genes in protein-protein interaction (PPI) networks, Shi et al. [41] identified potential miRNA-disease associations by performing random walk on the PPI network. Meanwhile, Mørk et al. [42] proposed the computational model of miRPD (miRNA-Protein-Disease), in which they did network analysis on both of the known protein-miRNA associations and the text mined disease-protein associations to infer miRNA–disease associations. However, these models also strongly relied on the interactions of miRNA and target with a high rate of false-positive results. MirAI model was proposed by Pasquier et al. [43] in which they represented different types of miRNA-related data, such as miRNA-disease associations information, miRNA-neighbor associations information, miRNA-target associations information, miRNA-word associations information and miRNA-family associations information, into a high-dimensionality vector space to further predicted the potential disease-miRNA association information. Obviously, the suitable choice of dimensionality was of great importance for the prediction performance. However, in their model there was no optimal dimension given. Recently, Chen et al. [44] proposed a Bipartite Network Projection for MiRNA–Disease Association prediction (BNPMDA) model based on integrated miRNA and disease similarity and the known miRNA–disease associations. They firstly defined the preference degree for miRNAs and diseases with the bias ratings. Then, bipartite network-based recommendation algorithm was implemented based on resource allocation process between miRNAs and diseases to predict the potential miRNA–disease associations.

Meanwhile, there also some other machine learning-based models be successively put forward later. For example, Chen et al. [45] developed the model of Regularized Least Squares for MiRNA-Disease Association (RLSMDA), which needed no negative samples resulting from the characteristic of semi-supervised learning. It’s worth pointing out that RLSMDA could be conducted for diseases without any known miRNA associations. Additionally, Xuan et al. [46] proposed a HDMP method to predict potential disease-miRNA associations based on weighted *k* most similar neighbors. In this model, they figured out the miRNA family and cluster information and recalculated miRNA functional similarity by endowing higher weight to miRNAs in the same family or cluster. However, the chosen number of neighbors would influence the prediction performance of the computational model to some extent. Considering that the traditional similarity-based k-nearest-neighbors (KNN) method was lazy learning and not reliable enough, Chen et al. [47] proposed a model of Ranking-based KNN for MiRNA-Disease Association prediction (RKNNMDA). In this model, to solve the limitation of normal ranking method, they firstly took use of SVM method via learning features from training data. Then, based on Hamming loss metric, they reranked the similarity-based sorted neighbors to obtain better prediction results. Furthermore, Chen et al. [48] proposed the first model that could infer the association types of disease-miRNA associations, namely the computational model of Restricted Boltzmann Machine for Multiple types of MiRNA-Disease Association prediction (RBMMMDA). It’s no doubt that the biology information about the different types of disease-miRNA associations obtained from RBMMMDA could benefit the understanding about the mechanism of diseases in the level of miRNAs. To further enhance the prediction performance, Chen et al. [49] then developed the model of Within and Between Score for MiRNA-Disease Association prediction (WBSMDA). This model was aimed to predict potential miRNAs related with plethora of human complex diseases by integrating the miRNA and disease Gaussian interaction profile kernel similarity, miRNA functional similarity, disease semantic similarity and also the known miRNA-disease associations. WBSMDA could also be utilized for new diseases and new miRNAs without any known relation information. Soon after, by integrating the biological dataset involved in WBSMDA into a heterogeneous graph, Chen et al. [50] further proposed another method named Heterogeneous Graph Inference for MiRNA-Disease Association prediction (HGIMDA). HGIMDA calculated the disease-miRNA association possibility by investigating all the 3-length paths in the constructed heterogeneous graph. HGIMDA obtained better prediction performance in terms of cross validation compared with most of previously mentioned models. Recently, Li et al. [51] presented a model of Matrix Completion for MiRNA-Disease Association prediction (MCMDA) using matrix completion algorithm to predict the potential miRNA-disease associations. In this model, they constructed initial matrix according to known miRNA-disease associations. Singular value threshold (SVT) algorithm was then implemented in the matrix completion process. The prediction scores were immediately calculated after they finished the matrix completion. By maximizing network information flow of the phenome-microRNAome network, Yu et al. [52] designed a combinatorial prioritization algorithm and proposed an computational model named MaxFlow to discover new disease-miRNA associations. Nowadays, Chen et al. [53] presented a model named Extreme Gradient Boosting Machine for MiRNA-Disease Association prediction (EGBMMDA), which was the first decision tree learning-based model for predicting novel miRNA–disease association. In this model, they constructed informative feature vector to train a regression tree under the gradient boosting framework built on the graph theoretical measures, statistical measures and matrix factorization outcomes for all the miRNA-disease pairs. Lately, in the literature review by Chen et al. [54] about miRNA-disease association prediction, 20 state-of-the-art in silico models were introduced from different perspectives. The authors summarized the existing difficulties in potential disease-miRNA association prediction task and pointed out five feasible and meaningful research schemas for further development of computational model designment in this field.

**Fig. 1:**
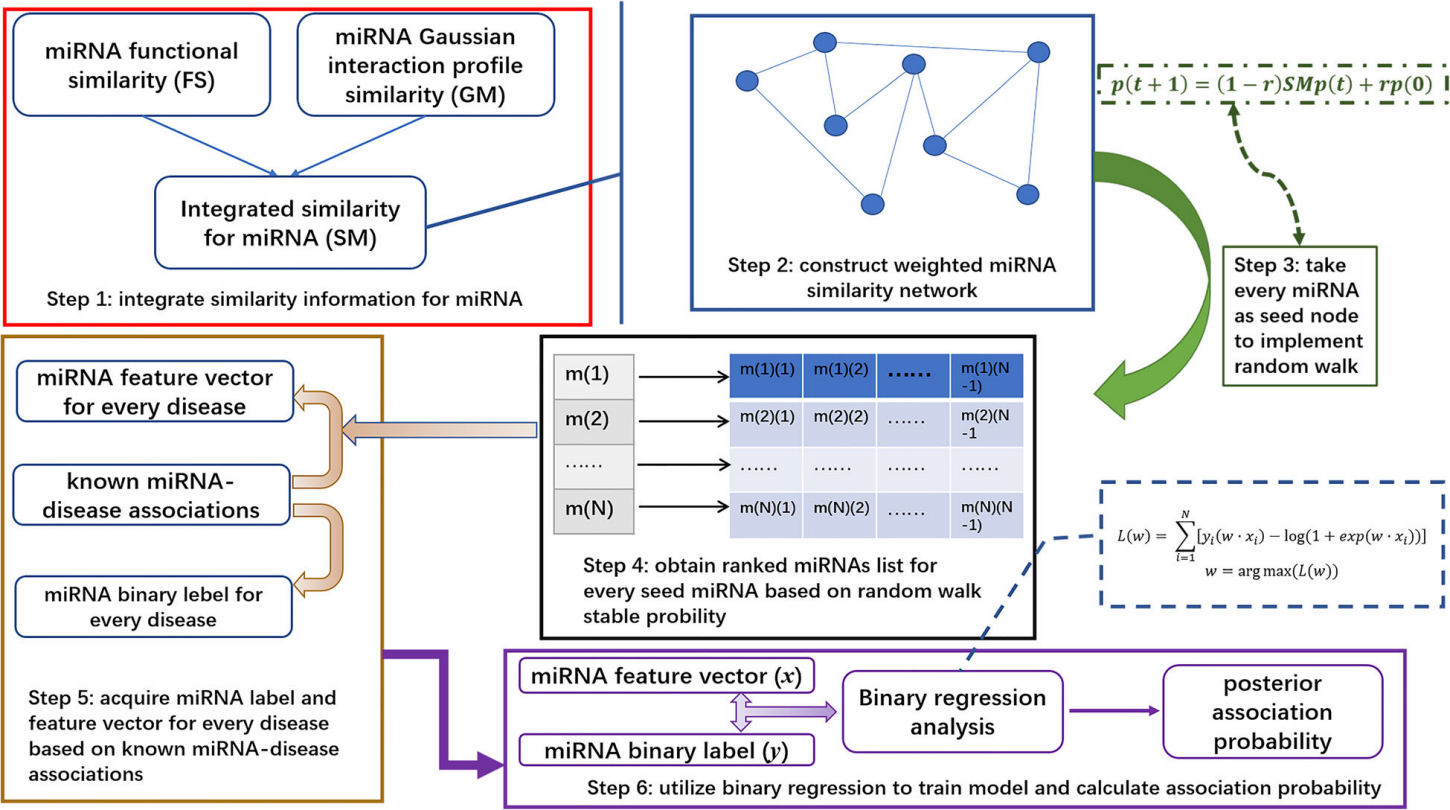
Flowchart of potential disease-miRNA association prediction based on the computational model of RWBRMDA

In this work, we presented a Random Walk and Binary Regression-based MiRNA-Disease Association prediction (RWBRMDA) method to predict underlying miRNA-disease associations. Specifically, we constructed an integrated miRNA similarity network based on miRNA functional similarity and miRNA Gaussian similarity. Then we implemented random walk with restart on the integrated miRNA similarity network for every miRNA in turn. Thirdly, we extracted feature vector for every miRNA according to the results of the random walk and the known miRNA-disease associations. Next, considering the field information about known disease-miRNA associations, we labelled 1 to those miRNAs with known associations with currently investigated disease, otherwise 0. Finally, we employed binary logistic regression method based on the feature vectors and label information to predict miRNAs for diseases of interest (See Fig. 1). Furthermore, we implemented Leave-one-out cross validation (LOOCV) for RWBRMDA. As a result, RWBRMDA obtained AUC value of 0.8076. What’s more, we carried out three different patterns of case studies in this work. Generally, in three types of case studies, we respectively evaluated the prediction performance of RWBRMDA for complex human diseases with miRNA associations recorded in HMDD v2.0 database [55], new diseases without any known related miRNAs and known diseases with miRNA associations recorded in HMDD v1.0 database [19]. By validating the prediction results based on other two important databases, miR2Disease [56] and dbDEMC [57], RWBRMDA obtained high confirmation ratios of the predicted miRNAs in all three ways of case studies. Therefore, it showed the effectivity of RWBRMDA in predicting potential miRNA-disease associations for various categories of diseases.

## Results

### Performance evaluation

**Fig. 2:**
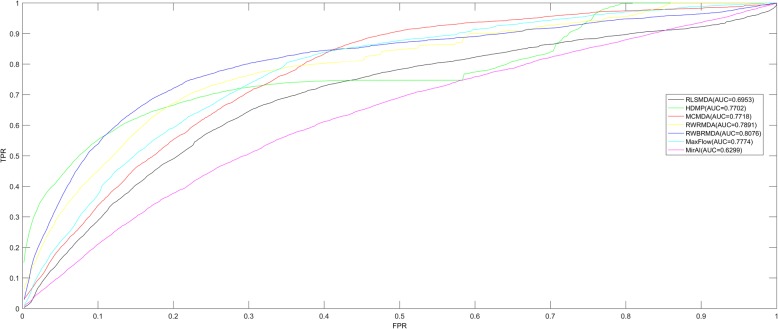
Performance comparisons between RWBRMDA and six state-of-the-art disease-miRNA association prediction models (RLSMDA, HDMP, MCMDA, RWRMDA, MaxFlow and MirAI) in terms of ROC curve and AUC value on LOOCV based on the same database of HMDD v2.0. As a result, RWBRMDA achieved AUC of 0.8076, which represented more outstanding prediction performance than the other mentioned models

Leave-one-out cross validation (LOOCV) is often utilized to evaluate the prediction performance of computational model. In this work, LOOCV was implemented as follows: for an investigated disease, based on the records in HMDD v2.0 [55] database, each known disease-related miRNA was left out in turn as test sample and the other known disease-related miRNAs were regarded as seed samples. Then, current test sample and candidate samples, namely the miRNAs without known association with the investigated disease would be ranked according to the prediction score of the model. If the test sample was ranked above the given threshold, the model would be considered to successfully predict this miRNA–disease association. Further, Receiver operating characteristics (ROC) curve could be drawn by plotting the true positive rate (TPR) versus the false positive rate (FPR) at different thresholds. Generally, the area under the ROC curve (AUC) is calculated and utilized to evaluate the prediction performance. Specifically, AUC = 1 means the best prediction performance and AUC = 0.5 indicates a random performance. As a result, RWBRMDA obtained the AUC value of 0.8076, which was higher than some previously mentioned computational models (RLSMDA: 0.6953 [45], HDMP:0.7702 [46], MCMDA:0.7718 [51], RWRMDA:0.7891 [40], MaxFlow:0.7774 [52], MirAI:0.6299 [43]) as shown in Fig. 2. It should be mentioned that we repeated all the 6 comparison methods based on the same HMDD v2.0 database, drew the corresponding ROC curves and compared the AUC values. In particular, the AUC value of MirAI seemed relatively small because the collaborative filtering technology utilized in this model was influenced by the sparsity problem of the biological data. Therefore, to some extent, RWBRMDA obtained better performance in the prediction of potential miRNA-disease associations.

### Case studies

As mentioned before, we carried out three different patterns of case studies in this work. Specifically, one approach was that we implemented RWBRMDA for disease-miRNA associations prediction based on the known diseases-miRNAs associations recorded in HMDD v2.0 database [55], then we verified the prediction results based on another two important miRNA-disease association databases, miR2Disease [56] and dbDEMC database [57]. The second approach was that we removed all the original miRNA associations information of the investigated disease, and then we verified the prediction results of the disease based on HMDD v2.0 database, miR2Disease and dbDEMC database. This method aimed to test the prediction performance for a new disease without any known associations. The third approach was that we used the diseases-miRNAs associations recorded in HMDD v1.0 database [19], then we verified the prediction results of some complex diseases based on HMDD v2.0 database, miR2Disease, and dbDEMC database. This approach aimed to assess the prediction robustness on different datasets of the computational model.

Case studies on Esophageal cancer and Prostate cancer were implemented in the first way. Esophageal cancer is a kind of cancer arising from the esophagus and it was reported as the sixth deadly cancers and the eighth most common cancer worldwide [58]. Statistical analysis showed that it was three to four times more common in male than female [59]. The treatment on esophageal cancer is strongly dependent on the cancer’s stage. There was clinic research showing that the survival rate could increase to 90% if the tumors could be diagnosed at an early stage [60]. Therefore, it’s obvious that the early detection of esophageal cancer is vital to cancer treatment [61, 62]. Some miRNAs have been confirmed to be related with esophageal cancer. For example, the relative expressions of miRNA-155, miRNA-183, and miRNA-20a in esophageal tissue were found to be significantly associated with increased risk for esophageal cancer [63]. In the case study for esophageal cancer, candidate miRNAs, namely miRNAs without known association with esophageal cancer in HMDD v2.0 database, were prioritized according to the scores obtained from RWBRMDA. As a result, 10 out of top 10, 47 out of top 50 were confirmed by recent experimental results recorded in miR2Disease and dbDEMC (See Table 1).

**Table 1:** We implemented RWBRMDA on esophageal cancer for potential disease-related miRNA prediction and conducted the first pattern of case study, in which the disease-miRNA associations recorded in HMDD v2.0 were used as training samples and miRNAs without known associations with currently considered diseases were regarded as test samples. According to the prediction results, among the top 10 and 50 potential esophageal cancer related miRNAs, 10 and 47 were confirmed by miR2Disease and dbDEMC databases

miRNA	Evidence	miRNA	Evidence
hsa-mir-29b	dbDEMC	hsa-mir-18a	dbDEMC
hsa-mir-1	dbDEMC	hsa-mir-221	dbDEMC
hsa-mir-19b	dbDEMC	hsa-mir-7	dbDEMC
hsa-mir-16	dbDEMC	hsa-mir-15b	dbDEMC
hsa-let-7e	dbDEMC	hsa-mir-106a	dbDEMC
hsa-mir-29a	dbDEMC	hsa-mir-218	unconfirmed
hsa-let-7d	dbDEMC	hsa-mir-10b	dbDEMC
hsa-mir-106b	dbDEMC	hsa-mir-132	dbDEMC
hsa-mir-146b	dbDEMC	hsa-mir-30c	dbDEMC
hsa-mir-222	dbDEMC	hsa-mir-429	dbDEMC
hsa-mir-24	dbDEMC	hsa-mir-93	dbDEMC
hsa-mir-200b	dbDEMC	hsa-mir-199b	dbDEMC
hsa-mir-181b	dbDEMC	hsa-mir-124	dbDEMC
hsa-let-7f	unconfirmed	hsa-mir-107	dbDEMC, miR2Disease
hsa-mir-181a	dbDEMC	hsa-mir-133b	dbDEMC
hsa-let-7i	dbDEMC	hsa-mir-23b	dbDEMC
hsa-mir-9	dbDEMC	hsa-mir-127	dbDEMC
hsa-let-7 g	dbDEMC	hsa-mir-206	dbDEMC
hsa-mir-125b	dbDEMC	hsa-mir-20b	dbDEMC
hsa-mir-17	dbDEMC	hsa-mir-122	unconfirmed
hsa-mir-195	dbDEMC	hsa-mir-224	dbDEMC
hsa-mir-142	dbDEMC	hsa-mir-18b	dbDEMC
hsa-mir-182	dbDEMC	hsa-mir-27b	dbDEMC
hsa-mir-30a	dbDEMC	hsa-mir-373	dbDEMC, miR2Disease
hsa-mir-125a	dbDEMC	hsa-mir-302b	dbDEMC

Prostate cancer develops in the epithelial cells of prostate, the cancer cells of which might spread from the prostate to other parts of the body, particularly the bones and lymph nodes [64]. Prostate cancer was reported to be the second leading cause of cancer-related death among men in developed countries [65]. Up to now, lots of miRNAs have been confirmed to be related to prostate cancer. For instance, it was reported that miRNA-183 expression was significantly higher in prostate cancer cells and tissues, compared with that in matched normal prostate cells and tissues [66]. It meant that the inhibition of miRNA-183 expression might be therapeutically beneficial for prostate cancer treatment [66]. Taking prostate cancer as a case study to implement RWBRMDA for potential miRNA-disease association prediction, for the top 10 and top 50 potential prostate cancer associated miRNAs, 10 and 45 of them were respectively confirmed to have experimental literature evidences recorded in miR2Disease and dbDEMC database (See Table 2). For example, miRNA-29b was ranked the second by RWBRMDA and it was the highest ranked miRNA, simultaneously confirmed by both miR2Disease and dbDEMC database. In fact, miRNA-29b was down-regulated from research about miRNA expression profiling of prostate cancer cell lines [67].

**Table 2:** We also conducted the first pattern of case study on prostate cancer by RWBRMDA. As a result, among the top 10 and 50 potential prostate cancer related miRNAs, 10 and 45 were confirmed by miR2Disease and dbDEMC databases

miRNA	Evidence	miRNA	Evidence
hsa-mir-146a	miR2Disease	hsa-mir-34a	dbDEMC, miR2Disease
hsa-mir-29b	dbDEMC, miR2Disease	hsa-mir-34c	dbDEMC
hsa-mir-1	dbDEMC	hsa-mir-200b	unconfirmed
hsa-mir-223	dbDEMC, miR2Disease	hsa-mir-155	dbDEMC
hsa-mir-21	dbDEMC, miR2Disease	hsa-mir-181b	dbDEMC, miR2Disease
hsa-mir-126	dbDEMC, miR2Disease	hsa-let-7f	dbDEMC, miR2Disease
hsa-let-7a	dbDEMC, miR2Disease	hsa-mir-133a	dbDEMC
hsa-let-7b	dbDEMC, miR2Disease	hsa-mir-181a	dbDEMC, miR2Disease
hsa-mir-19b	dbDEMC, miR2Disease	hsa-mir-196a	dbDEMC
hsa-mir-29c	dbDEMC	hsa-let-7i	dbDEMC
hsa-let-7c	dbDEMC, miR2Disease	hsa-mir-9	dbDEMC
hsa-mir-16	dbDEMC, miR2Disease	hsa-let-7 g	dbDEMC, miR2Disease
hsa-mir-15a	dbDEMC, miR2Disease	hsa-mir-34b	dbDEMC
hsa-mir-143	dbDEMC, miR2Disease	hsa-mir-17	miR2Disease
hsa-let-7e	dbDEMC	hsa-mir-203	unconfirmed
hsa-mir-29a	dbDEMC, miR2Disease	hsa-mir-195	dbDEMC, miR2Disease
hsa-mir-199a	dbDEMC, miR2Disease	hsa-mir-205	dbDEMC, miR2Disease
hsa-mir-150	dbDEMC	hsa-mir-142	unconfirmed
hsa-let-7d	dbDEMC, miR2Disease	hsa-mir-182	dbDEMC, miR2Disease
hsa-mir-106b	dbDEMC	hsa-mir-30a	miR2Disease
hsa-mir-210	miR2Disease	hsa-mir-101	dbDEMC, miR2Disease
hsa-mir-146b	unconfirmed	hsa-mir-200a	dbDEMC
hsa-mir-222	dbDEMC, miR2Disease	hsa-mir-19a	dbDEMC
hsa-mir-24	dbDEMC, miR2Disease	hsa-mir-125a	dbDEMC, miR2Disease
hsa-mir-200c	dbDEMC	hsa-mir-18a	unconfirmed

We conducted case study on Breast cancer by way of the second case study method, in which we removed all the related miRNAs information of breast cancer to model the situation where a new disease without known miRNA associations was investigated. Breast cancer is known as the most leading type of cancer in women worldwide, accounting for about 25% of all the female’s death cases all over the world [68]. Some researches on breast cancer have confirmed that many miRNAs were associated with breast cancer. For example, microarray-based miRNA profiling on whole blood of early stage breast cancer patients showed that miRNA-106b was up-regulated in whole blood of breast cancer patients [69]. What’s more, it was found that downregulation of miRNA-140 promoted cancer stem cell formation in basal-like early stage breast cancer [70]. We verified the predicted underlying breast cancer related miRNAs obtained by RWBRMDA. Consequently, 10 out of the top 10 and 49 out of the top 50 predicted miRNAs were experimentally confirmed by HMDD v2.0, miR2Disease and dbDEMC database (See Table 3).

**Table 3:** We conducted case study of breast cancer in the second way by RWBRMDA, in which we removed all the known breast cancer related miRNAs to simulate a new disease without any known associations. Then we verified the prediction results based on HMDD v2.0 database, miR2Disease and dbDEMC database. As a result, among the top 10 and 50 potential miRNAs, 10 and 49 were confirmed

miRNA	Evidence	miRNA	Evidence
hsa-mir-146a	dbDEMC,miR2Disease,HMDD v2.0	hsa-mir-146b	dbDEMC,miR2Disease,HMDD v2.0
hsa-mir-21	dbDEMC,miR2Disease,HMDD v2.0	hsa-mir-221	dbDEMC,miR2Disease,HMDD v2.0
hsa-mir-1	dbDEMC,HMDD v2.0	hsa-mir-210	dbDEMC,miR2Disease,HMDD v2.0
hsa-mir-29b	dbDEMC,miR2Disease,HMDD v2.0	hsa-mir-24	dbDEMC,HMDD v2.0
hsa-let-7a	dbDEMC,miR2Disease,HMDD v2.0	hsa-mir-200b	dbDEMC,miR2Disease,HMDD v2.0
hsa-mir-155	dbDEMC,miR2Disease,HMDD v2.0	hsa-mir-106b	dbDEMC,HMDD v2.0
hsa-mir-145	dbDEMC,miR2Disease,HMDD v2.0	hsa-mir-18a	dbDEMC,miR2Disease,HMDD v2.0
hsa-mir-223	dbDEMC,HMDD v2.0	hsa-mir-181a	dbDEMC,miR2Disease,HMDD v2.0
hsa-mir-126	dbDEMC,miR2Disease,HMDD v2.0	hsa-mir-34c	dbDEMC,HMDD v2.0
hsa-mir-19b	dbDEMC,HMDD v2.0	hsa-mir-19a	dbDEMC,HMDD v2.0
hsa-let-7b	dbDEMC,HMDD v2.0	hsa-mir-181b	dbDEMC,miR2Disease,HMDD v2.0
hsa-mir-29a	dbDEMC,HMDD v2.0	hsa-mir-200c	dbDEMC,miR2Disease,HMDD v2.0
hsa-mir-16	dbDEMC,HMDD v2.0	hsa-let-7f	dbDEMC,miR2Disease,HMDD v2.0
hsa-let-7c	dbDEMC,HMDD v2.0	hsa-mir-133a	dbDEMC,HMDD v2.0
hsa-mir-17	miR2Disease,HMDD v2.0	hsa-mir-196a	dbDEMC,miR2Disease,HMDD v2.0
hsa-mir-15a	dbDEMC,HMDD v2.0	hsa-let-7i	dbDEMC,miR2Disease,HMDD v2.0
hsa-mir-29c	dbDEMC,miR2Disease,HMDD v2.0	hsa-let-7 g	dbDEMC,HMDD v2.0
hsa-mir-34a	dbDEMC,HMDD v2.0	hsa-mir-203	dbDEMC,miR2Disease,HMDD v2.0
hsa-mir-143	dbDEMC,miR2Disease,HMDD v2.0	hsa-mir-142	unconfirmed
hsa-let-7e	dbDEMC,HMDD v2.0	hsa-mir-9	dbDEMC,miR2Disease,HMDD v2.0
hsa-mir-150	dbDEMC	hsa-mir-195	dbDEMC,miR2Disease,HMDD v2.0
hsa-mir-199a	dbDEMC,HMDD v2.0	hsa-mir-205	dbDEMC,miR2Disease,HMDD v2.0
hsa-mir-125b	miR2Disease,HMDD v2.0	hsa-mir-92a	HMDD v2.0
hsa-mir-222	dbDEMC,miR2Disease,HMDD v2.0	hsa-mir-34b	dbDEMC,HMDD v2.0
hsa-let-7d	dbDEMC,miR2Disease,HMDD v2.0	hsa-mir-182	dbDEMC,miR2Disease,HMDD v2.0

Lymphoma often refers to a group of cancerous blood cell tumors that developed from lymphocytes [71]. Worldwide, lymphoma was reported to be the seventh-most common cancer and also be the third-most common cancer in children [72]. Benefitting from the development of deep sequencing technology, several miRNAs have been discovered to be related with lymphomas. For example, miRNA-155, miRNA-21 and miRNA-221 were observed over-expressed in lymphoma cell lines [73]. In order to test the prediction robustness of RWBRMDA in different datasets, we conducted the third way of case study on lymphoma, in which we only used the known disease-related miRNAs recorded in HMDD v1.0 database as training samples and used associations in HMDD v2.0 database, miR2Disease, and dbDEMC database as test datasets. As a result, 10 out of the top 10 and 44 out of the top 50 predicted miRNAs were confirmed based on the three test datasets (See Table 4). For instance, miRNA-29c, which was the highest ranked miRNA confirmed by dbDEMC and HMDD v2.0 databases, was reported to show down-regulation in lymphoma cells [74] .

**Table 4:** Lymphoma was conducted as a case study in the third way, in which we only used known disease-miRNA association based on HMDD v1.0 database as test samples to assess the robustness of the prediction model in the different datasets, and then we verified the prediction results according to HMDD v2.0 database, miR2Disease, and dbDEMC database. As a result, among the top 10 and 50 potential lymphoma related miRNAs, 10 and 44 were confirmed

miRNA	Evidence	miRNA	Evidence
hsa-mir-223	dbDEMC	hsa-mir-200b	dbDEMC,HMDD v2.0
hsa-mir-15b	dbDEMC	hsa-mir-199a	dbDEMC
hsa-mir-29c	dbDEMC,HMDD v2.0	hsa-mir-95	dbDEMC
hsa-mir-106a	dbDEMC	hsa-mir-183	dbDEMC
hsa-mir-146a	dbDEMC,HMDD v2.0	hsa-let-7e	dbDEMC
hsa-mir-99b	dbDEMC	hsa-mir-141	dbDEMC
hsa-mir-100	dbDEMC	hsa-let-7c	dbDEMC
hsa-mir-145	dbDEMC	hsa-let-7a	dbDEMC
hsa-mir-143	dbDEMC	hsa-mir-128b	unconfirmed
hsa-mir-155	dbDEMC,HMDD v2.0	hsa-mir-21	dbDEMC,HMDD v2.0
hsa-mir-222	dbDEMC	hsa-mir-29b	dbDEMC
hsa-let-7 g	dbDEMC	hsa-mir-34c	unconfirmed
hsa-mir-101	dbDEMC,HMDD v2.0	hsa-mir-214	dbDEMC
hsa-mir-224	dbDEMC	hsa-mir-127	dbDEMC
hsa-mir-34a	dbDEMC	hsa-let-7b	dbDEMC
hsa-mir-146b	unconfirmed	hsa-mir-132	dbDEMC
hsa-mir-221	dbDEMC	hsa-mir-137	dbDEMC
hsa-mir-125b	unconfirmed	hsa-mir-376c	unconfirmed
hsa-let-7i	dbDEMC	hsa-mir-181b	dbDEMC
hsa-mir-203	dbDEMC,HMDD v2.0	hsa-mir-139	dbDEMC,HMDD v2.0
hsa-mir-126	dbDEMC,HMDD v2.0	hsa-mir-122	dbDEMC,HMDD v2.0
hsa-mir-335	dbDEMC	hsa-mir-31	dbDEMC
hsa-mir-196b	unconfirmed	hsa-mir-9	dbDEMC
hsa-mir-140	dbDEMC	hsa-mir-181d	dbDEMC
hsa-mir-191	dbDEMC	hsa-mir-206	dbDEMC

In conclusion, the promising results obtained from LOOCV and case studies in three different ways have demonstrated the reliable prediction performance of RWBRMDA. Therefore, we further prioritized candidate miRNAs for all the diseases recorded in HMDD v2.0 database. The predicted ranks of miRNAs for each disease were publicly released for further experimental validation (Additional file 1). The potential disease-miRNA associations with relatively high ranks were expected to be confirmed by clinical observation or biological experiments in the future.

## Discussion

Several important factors contributed to the excellent performance of RWBRMDA. Firstly, benefitting from the valid and updated disease-miRNA association data by abundant biology researches, RWBRMDA could have more chance to obtain higher prediction accuracy. Secondly, RWBRMDA took full advantage of the similarity information of the miRNA functional similarity and Gaussian interaction profile kernel similarity to obtain integrated global similarity network for miRNAs. Generally, the more similarity information was utilized, the better prediction performance would be. Thirdly, based on the previously mentioned similarity information, RWBRMDA further implemented random walk with restart, an effective and widely used method, to investigate global reachability between any pair of miRNAs. A higher stable probability meant a higher reachability between two miRNAs or meant a higher association probability with the same disease of these two miRNAs. Then according to the random walk result we could extract more reliable feature vector for every miRNA as the input of next binary logistic regression. More reliable and valuable feature vector would be help for a better output of the binary logistic regression. In other words, integrating random walk and binary logistic regression was an innovative and efficient research practice.

There were also some limitations in RWBRMDA. Firstly, because we took use of binary logistic regression in the model, we needed prior association label information for investigated miRNAs. If the known association information was too little or none, the AUC value of RWBRMDA might be a little lower. Secondly, RWBRMDA partly depended on the parameters used in our model, such as the restart probability in random walk and the length of the feature vector of miRNA. Hence, a technical analysis for selecting appropriate and optimized parameter values was necessary when RWBRMDA was conducted based on other biology dataset.

## Conclusions

Identifying potential miRNA-disease associations was vitally important for investigating the biomarker of disease diagnosis at the miRNA level. Based on the fundamental hypothesis that functionally similar miRNAs greatly tended to be relevant to phenotypically similar diseases and vice versa, in this work, we introduced a computational model named RWBRMDA to predict underlying miRNA-disease associations. RWBRMDA was developed mainly based on random walk with restart and binary logistic regression. The known miRNA-disease association information in HMDD v2.0 database was utilized to assign prior label to miRNAs for any disease we investigated. Considering that the network modeling was a primitive and intuitive way for modeling biological data, we also took use of miRNA functional similarity, Gaussian interaction profile kernel similarity for miRNAs and integrated similarity for miRNAs to map miRNAs to a weighted network. We complemented random walk with restart on the constructed network for every miRNA to obtain the global feature vector of miRNA, which was used for binary logistic regression with the known prior label information to calculate the posterior association probability of investigated disease-miRNA pairs (See Fig. 1). Both cross validation result (AUC = 0.8076) and three different kinds of case study on esophageal cancer (94%), prostate cancer (90%), breast cancer (98%) and lymphoma (88%) have demonstrated the reliable prediction ability of RWBRMDA. Therefore, RWBRMDA was anticipated to be valuable for further research on miRNA-disease associations and be beneficial to human disease diagnosis, treatment, prevention and prognosis.

## Methods

### Human miRNA-disease associations

In this study, we take use of human disease-miRNA associations in HMDD v2.0 database [55], which records 5430 known miRNA-disease associations with respect to 383 human diseases and 495 miRNAs. Technically, we could construct the adjacent matrix *A* to clearly describe the relation of each disease-miRNA pairs. Specifically, if miRNA *m(i)* is confirmed to be related to disease *d(j)* in the database, the entry *A(i,j)* is defined as 1, otherwise 0. Finally, 5430 entries of matrix *A* are assigned 1, the rest ones are assigned 0.

### MiRNA functional similarity

Based on the basic assumption that miRNAs with similar function are more likely to be related to semantically similar diseases and vice versa, miRNA functional similarity have been calculated by Wang et al [32]. In our study, owning to their relevant researches, we obtain the miRNA functional similarity information from http://www.cuilab.cn/files/images/cuilab/misim.zip. Furthermore, we construct the miRNA functional similarity matrix *FS* to store the data, where the entry *FS(i,j)* describes the functional similarity between miRNA *m(i)* and miRNA *m(j)*.

### Gaussian interaction profile kernel similarity

Thanks to a kind of widely used Gaussian kernel function, which named Radial Basis Function (RBF), Gaussian interaction profile kernel similarity could be calculated and put into use for prediction task [75]. The interaction profile of miRNA *m(i)* could be expressed built on the adjacency matrix *A*. Specifically, based on the binary vector *IP(m(i))*, namely the *ith* row of the adjacency matrix *A*, Gaussian kernel similarity between miRNA *m(i)* and *m(j)* could be obtained:

1 $$ GM\left(m(i),m(j)\right)=\mathit{\exp}\left(-{\gamma}_m{\left\Vert IP\left(m(i)\right)- IP\left(m(j)\right)\right\Vert}^2\right) $$

where *r*_*m*_ is used to control bandwidth of the kernel, *GM* is denoted as the Gaussian interaction profile kernel similarity matrix for miRNAs. What’s more, *r*_*m*_ could be calculated by normalizing a new bandwidth parameter ${r}_m^{\prime }$ by the average number of known associations with diseases per miRNA as follows:

2 $$ {\upgamma}_{\mathrm{m}}=\frac{\upgamma_{\mathrm{m}}^{\prime }}{\left(\frac{1}{n_m}{\sum}_{\mathrm{i}=1}^{n_m}{\left\Vert IP\left(m\left(\mathrm{i}\right)\right)\right\Vert}^2\right)} $$

where *n*_*m*_ is the number of all the miRNAs investigated. In this article, ${r}_m^{\prime }$ is set 1 based on previous studies [76, 77].

### Integrated similarity for miRNAs

Integrated miRNA similarity between miRNAs *m(i)* and *m(j)* is calculated based on the miRNA functional similarity and Gaussian interaction profile kernel similarity for miRNA [49] as follows, and *SM* is defined as the integrated miRNA similarity matrix:

3 $$ SM\left(m(i),m(j)\right)=\left\{\begin{array}{c} FS\left(m(i),m(j)\right)\ m(i)\  and\ m(j)\  has\  functional\ similarity\\ {} GM\left(m(i),m(j)\right)\  otherwise\ \end{array}\ \right. $$

### RWBRMDA

In this work, we propose a computational model of RWBRMDA by integrating known miRNA-disease associations, miRNA functional similarity and Gaussian interaction profile kernel similarity for miRNAs (See Fig. 1) motivated by study in [78, 79]. It’s known that random walk could be used to rank the relation probability for the nodes in a network. Binary regression could be used for classification problems or prediction problems. We implement random walk with restart for every miRNA on the integrated miRNA similarity network to obtain corresponding feature vector of the investigated miRNA. Based on the feature vectors of miRNAs and the known miRNA-disease associations, we could assign the binary label 0 or 1 to every miRNA for the given disease. Then we utilize binary regression to predict the association probability between the miRNA with label 0 and the corresponding disease of interest.

Technically, based on the known miRNA-disease associations in HMDD v2.0, we have constructed the adjacent matrix *A.* According to the integrated similarity matrix *SM* for miRNAs, we construct a weighted miRNAs relation network, which consists of 495 miRNA nodes. The weight of pairwise miRNAs in the network is assigned their integrated similarity value in the *SM*. Random walk with restart is then implemented on the weighted network, taking every miRNA node as start node in turn. Specifically, every miRNA node is considered as seed node for one time of random walk with restart. Other miRNA nodes are considered as candidate nodes. For a seed miRNA *m(i)*, the initial probability *p(0)* is the normalized *ith* row of matrix *SM*. Here we define the restart probability of random walk at source nodes as *r* (0 < *r* < 1) in every time step. Then a vector *p(t)* could be defined in which the *jth* element meant the probability of finding the walker at node *j* at step *t*. Finally, the random walk process could be defined as follows:

4 $$ p\left(t+1\right)=\left(1-r\right) SMp(t)+ rp(0) $$

Random walk would finally reach the stable state after some steps. We call the random walk reach the stationary stage if the change between *p(t)* and *p(t + 1)* is less than a cutoff (here we chose 10^− 6^ as the cutoff) measured by *L1* norm. When the random walk reaches the stable state, the candidate miRNAs for the seed miRNA *m(i)* could be ranked built on the stable probability of *p*_∞_(*m*(*i*)). Generally, after 495 times random walk on the weighted miRNAs relation network, we could obtain the corresponding ranked candidate miRNAs sequence or list for every seed miRNA, which we call the global relationship information of every miRNA. In previous model of RWRMDA [40], the author also uses random walk with restart. While, there exists many differences between the implementation progress of our model and the implementation progress of RWRMDA model. First, the motivation of random walk is different. In RWRMDA, random walk is used directly to predict disease-related miRNAs, which means they aim to mine the pair-wise relationship of miRNA and disease. In current work of RWBRMDA, we utilize random walk to seek the relationship between miRNAs, which is more suitable because the random walk process is conducted on the miRNA similarity network. Second, the choices of seed nodes are different. In RWRMDA, they choose known disease-related miRNAs as seed nodes, while in this work we take every miRNA in turn as seed node, which is more practical in cases where the field knowledge is short. In principle, the aims of random walk in these two works were different.

Next, for an arbitrary disease *d(j),* the *j*th column of adjacency matrix *A* is regarded as the binary label vector of all the miRNAs with respect to disease *d(j)*. Binary logistic regression is then conducted to calculate the posterior association probability of those miRNAs with label 0 to *d(j)* as follows:

5 $$ \mathrm{P}\left(y=1|x\right)=\frac{\mathit{\exp}\left(w\bullet x\right)}{1+\mathit{\exp}\left(w\bullet x\right)} $$

6 $$ \mathrm{P}\left(y=0|x\right)=\frac{1}{1+\mathit{\exp}\left(w\bullet x\right)} $$

where *y* is the binary label, *w* is the weight vector, which needs to be trained, *w* ∙ *x* is the inner product of vector *w* and vector *x*.

Given a training set of T = {(*x*_1_, *y*_1_), (*x*_2_, *y*_2_), …(*x*_*N*_, *y*_*N*_)}, where *x*_*i*_ ∈ *R*^*n*^, *y*_*i*_ ∈ {0, 1} and *N* is the number of samples, we could train the parameter *w* by maximum likelihood estimator. Likelihood function is calculated as follows:

7 $$ {\prod}_{i=1}^N{\left[\pi \left({x}_i\right)\right]}^{y_i}{\left[1-\pi \left({x}_i\right)\right]}^{1-{y}_i} $$

where $\pi \left({x}_i\right)=\mathrm{P}\left(y=1|{x}_i\right)=\frac{\mathit{\exp}\left(w\bullet {x}_i\right)}{1+\mathit{\exp}\left(w\bullet {x}_i\right)}$, maximum likelihood function means maximizing the following logarithm function, namely

8 $$ L(w)={\sum}_{i=1}^N\left[{y}_i\mathit{\log}\pi \left({x}_i\right)+\left(1-{y}_i\right)\mathit{\log}\left(1-\pi \left({x}_i\right)\right)\right] $$

then we could obtain:

9 $$ L(w)={\sum}_{i=1}^N\left[{y}_i\left(w\bullet {x}_i\right)-\mathit{\log}\left(1+\mathit{\exp}\left(w\bullet {x}_i\right)\right)\right] $$

10 $$ w=\arg \max \left(L(w)\right) $$

Suppose the maximum likelihood estimation for *w* is *w*^∗^, then the binary logistic regression model finally becomes:

11 $$ \mathrm{P}\left(y=1|x\right)=\frac{\mathit{\exp}\left({w}^{\ast}\bullet x\right)}{1+\mathit{\exp}\left({w}^{\ast}\bullet x\right)} $$

12 $$ \mathrm{P}\left(y=0|x\right)=\frac{1}{1+\mathit{\exp}\left({w}^{\ast}\bullet x\right)} $$

Back to our prediction task for the novel miRNA-disease associations, the *j*th column of matrix *A* is regarded as the binary label vector of all the miRNAs with respect to disease *d(j).* If we could find feature vector for every miRNA with respect to disease *d(j)*, we could then utilize binary logistic regression model to calculate the association probability for miRNAs with label 0 to disease *d(j)*. Certainly, previously descripted random walk strategy is prepared for extracting feature vector for miRNAs. Assume we have already performed random walk with restart for miRNA *m(i)* on the weighted integrated miRNAs network and gotten the global relationship information for miRNA *m(i)*, namely the candidate miRNA ranks for seed miRNA *m(i).* Here to extract feature vector of *m(i)*, we consider the top *K* ranked candidate miRNAs according to the random walk result. In this work, *K* is considered as 50, namely about 10% of the total number of miRNAs. These top *K* ranked candidate miRNAs would be used to build feature vector of *m(i)*. For a disease *d*, the feature vector of *m(i)* with respect to *d* is regarded as *Vec(m(i))*, as follows:

13 $$ Vec\left(m(i)\right)=\left(1,{\varnothing}_{i1},{\varnothing}_{i0}\right) $$

where the stable random walk probability of the top *K* ranked candidate miRNAs with label 1 respect to disease *d* were added up as∅_*i*1_. Similarly, we added up the stable random walk probability of the top *K* ranked miRNAs with label 0 respect to disease *d* as ∅_*i*0_. Specially, the element ‘1’ in the feature vector represents the constant term. Then for disease *d*, we get the ternary feature vector of every miRNA. Together with the binary label information and feature vector of miRNA, we could easily take use of binary logistic regression model to calculate the posterior association probability for the given disease.

## Additional file

Additional file 1: We prioritized corresponding candidate miRNAs for all the diseases recorded in HMDD v2.0 database. The predicted results for each disease were publicly released for further experimental validation. The potential and promising disease-miRNA associations with relatively high ranks were expected to be confirmed by biological experiments and clinical observation in the future. (XLSX 4198 kb)

## Abbreviations

RWR: random walk with restart
ROC: receiver operating characteristics
AUC: the area under the ROC curve
LOOCV: leave-one-out cross validation
RBF: radial basis function
TPR: true positive rate
FPR: false positive rate
HMDD: human microRNA disease database
dbDEMC: database of differentially expressed miRNAs in human cancers
